# Low-cost solar-powered urban soundscape sensor

**DOI:** 10.1016/j.ohx.2026.e00753

**Published:** 2026-02-28

**Authors:** Lion Cassens, Maarten Kroesen, Simeon Calvert, Sander van Cranenburgh

**Affiliations:** a Delft University of Technology, Faculty of Technology, Policy and Management, Jaffalaan 5, 2628 BX Delft, the Netherlands; b Delft University of Technology, Faculty Of Civil Engineering and Geosciences, Stevinweg 1, 2628 CN Delft, the Netherlands

**Keywords:** Noise pollution, Soundscape, Edge AI

## Abstract

Noise pollution negatively affects health and well-being, making its monitoring important for effective mitigation strategies. Sensor systems such as sound level meters have long been used for this purpose. Nevertheless, dependence on grid power, restricted metrics beyond loudness, and high costs per unit limit current solutions. This paper presents an open hardware, off-grid sound sensor to measure loudness and complementary noise metrics. The sensor detects eleven common urban sound events, calculates acoustic sharpness, and the intermittency ratio of the acoustic environment. The sensor is based on an ESP32-S3 microcontroller on a customized printed circuit board, optimized to address the current limitations. The board includes a battery management circuit for solar charging, a real-time clock for accurate time keeping, and supports LoRaWAN to send aggregated metrics. The latter allows remote monitoring, while more detailed metrics are stored on a microSD card. A solar panel and up to two 18650 Li-Ion or LiFePo4 batteries allow the sensor to be deployed independently of mains power. The open hardware is accompanied by open firmware, which has been organized into multiple components to allow easy changes and extensions for other use cases. A lab validation showed a deviation below 2 dB for a 1 kHz test tone compared to a calibrated sound level meter.

## Graphical abstract

**Figure:**
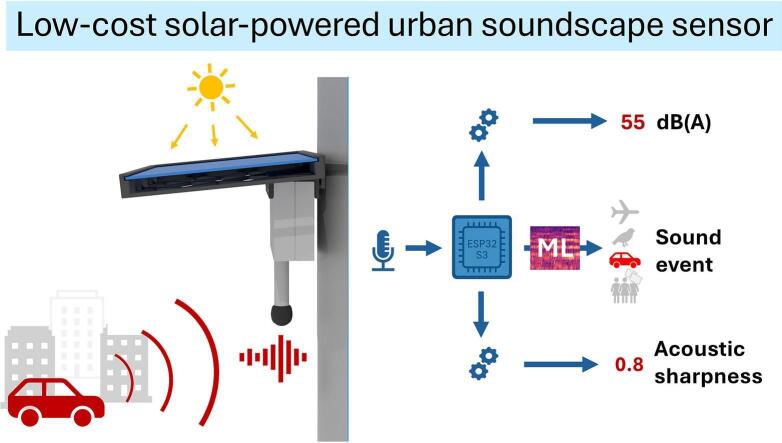

Specifications table.

**Table:** 

Hardware name	Low-cost solar-powered urban soundscape sensor
Subject area	• Environmental, planetary and agricultural sciences
Hardware type	• Field measurements and sensors
Closest commercial analog	No commercial analog is available.
Open source license	MIT License
Cost of hardware	Approximately EUR 55 unit cost, depending on the total quantity.
Source file repository	https://doi.org/10.17605/OSF.IO/VS4YZ

## 1 Hardware in context

Noise pollution is one of the most pervasive urban pollutants of our time. While bustling cities create vast societal and economic opportunities, they also create high levels of noise. Traffic is typically the most dominant, but by far not the only sound source [1]. The urban soundscape is also accompanied by chatter from crowds and nightlife, sirens, and music. Not only do man-made nuisances mask more pleasant acoustic features, such as bird song, but they also have serious health consequences. Noise pollution increases the risk for cardiovascular disease, can lead to hearing loss, and has been associated with diabetes [2]. In Europe, in just one year, noise pollution has been linked to 50,000 cases of cardiovascular disease and a loss of 1.3 million healthy life years [3]. Moreover, noise exposure may hinder cognitive development in children and lead to lower reading comprehension [4], [5].

The effects on health and well-being highlight the need to measure and monitor noise pollution for well-informed policies and urban planning. Traditional noise monitoring relies on calibrated and certified Sound Level Meters (SLM). While they remain the benchmark for legal compliance, the accuracy of these devices comes at a high cost: a calibrated handheld SLM costs between EUR 200 and EUR 5,000. Mountable SLMs with waterproofing and connectivity result in even higher expenses for unsupervised monitoring. This motivated researchers and practitioners to explore various low-cost alternatives to SLMs. The lower price point of “Do-It-Yourself” (DIY) sensors promises affordable large-scale deployments while also being open for custom implementation of noise metrics beyond pure loudness levels. In this work, the term sensor refers to a self-contained sound sensing node, comprising a microphone, processing unit, power supply, and communication interface for the purpose of unsupervised environmental noise monitoring.

Low-cost sound sensors typically use MEMS microphones, which are much more affordable than other capsules and still accurate enough for many noise monitoring use cases [6]. By now, many such systems have been described, both in scientific work and in grey literature [7], [8], [9], [10], [11], [12]. Some systems rely on powerful platforms like Raspberry Pi [9], [13]. Such miniature PCs make the development of the required software easier, but lead to substantial power consumption. The “Sounds of New York City” (SONYC) project is a good example of this: Not only does the sensor network measure loudness, but it also captures sound fragments and classifies the sound source [9]. In return, the SONYC sensors need to be plugged into mains power permanently. Other sensors rely on microprocessors without a full-fledged operating system and thus, are less power demanding [8], [10]. However, most of these low-power systems have been used only to calculate loudness levels – without an on-device classification of sound events. Existing systems also rely on off-the-shelf development boards. While these are convenient for rapid development, they serve a broader purpose and are thus not tailored specifically for environmental noise monitoring. This becomes apparent in the missing battery management or solar panel support of many sensors. Development boards are also often not optimized for energy efficiency and cost, as their primary purpose is fast prototyping and debugging [14]. Consequently, they may include auxiliary components, such as debugging interfaces, potentially increasing power consumption and expenses.

The limitations of existing solutions can be summarized into three clear gaps. Firstly, most microcontroller-based sound sensors support only limited sound metrics. On-device sound event detection is reserved for more expensive and demanding platforms. Secondly, power requirements often result in a dependency on mains power. This significantly increases the installation effort for large-scale urban sound monitoring and therefore diminishes the promised advantages over SLMs. And lastly, the costs for more capable sensor systems are still higher than necessary due to unnecessarily powerful, and thus expensive, off-the-shelf components.

The present work addresses these limitations with a reproducible open hardware and open firmware sensor system. This system contributes to future noise monitoring studies by lowering the deployment costs and power dependencies, while providing efficient sound source classifications alongside dB(A) measurements of loudness.

## 2 Hardware description

**Fig. 1:**
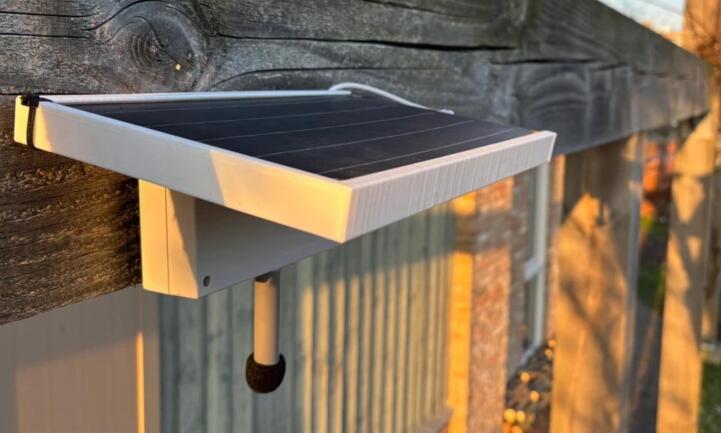
Deployed sensor system with sensor enclosure, solar panel, solar panel holder, and microphone.

The sensor system presented in this paper has been designed around the following three requirements: (1) Independence from mains power for flexible deployments, (2) low cost to facilitate large-scale deployments, and (3) support for sound metrics in addition to loudness, such as sound event classification and sharpness. The system consists of an enclosure holding most electronics and batteries, a microphone, and a holder that connects a solar panel to the enclosure (see Fig. 1).

Independence from mains power: The solar panel, in combination with up to two 18650 cells (Li-Ion or LiFePo4), eliminates the need for external power. With sufficient sunlight present, the solar panel directly powers the sensor. Excessive energy is stored in the battery, which acts as a buffer for the night and cloudy weather. If solar input remains insufficient over a prolonged period, the sensor will reduce the observation duration and periodically enter a low-power sleep mode. This power management feature prevents the battery from being fully depleted. The power management is described in more detail in Section 2.2: Firmware components. Moreover, the independence from mains power enables a quick installation on, e.g., light poles or balconies. Under- and over-discharge protection is present on the sensor PCB.

Cost and scalability: The PCBs and electronics (including the microphone) cost EUR 12 per unit. With assembly by common manufacturers (e.g., JLCPCB, PCBWay), fully populated PCBs cost approximately EUR 25 per sensor. The exact unit price depends on the number of units due to fixed setup fees. A complete sensor system, including a solar panel, housing, microSD card, and battery, costs around EUR 55. This is significantly cheaper than traditional sound level meters or Raspberry Pi-based systems. For comparison, a Raspberry Pi 4 with 2 GB RAM on its own costs about EUR 50. Traditional sound level meters are classified according to IEC 61672–1 [15] into Class 2 (tolerance of ±1.4 dB) and Class 1 (tolerance of ±1 dB) devices. Handheld Class 2 sound level meters are available starting at approximately EUR 170 [16]. For continuous environmental monitoring, however, a weather-resistant measurement station is required. Commercial Class 2 monitoring stations are listed at prices of around EUR 2500, while Class 1 stations are commonly listed between EUR 3000 and 6700 [17]. Prices are indicative and based on publicly available list prices at the time of writing. Overall, the general price range is significantly higher than that of the proposed sensor.

Noise metrics: The firmware currently supports the measurement of loudness in dB(A), a simplified version of psychoacoustic sharpness, and the detection of 11 common urban sound events, such as traffic noise, bird song, and music. Furthermore, Intermittency Ratios (IR) can be computed from the recorded dB(A) levels. The IR describes how much of the sound power is part of distinct, loud events instead of continuous background noise [18]. If required, additional metrics or machine learning models could be implemented by the user.

The sensor has been designed primarily for temporary research deployments with a duration of a few months. Detailed noise metrics are saved on a microSD card (e.g., dB(A) in a 125 ms resolution) and can be retrieved at the end of the deployment. Aggregated real-time data can be transmitted via LoRaWAN, which can be used for monitoring purposes. The sensor follows the LoRaWAN class A implementation. LoRaWAN has a much larger range than Wi-Fi. For reference, real-world LoRaWAN measurements indicated a range of up to 4.4 km in an urban environment [19]. Similar experiments for outdoor Wi-Fi show a range of up to 100–500 m [20]. Depending on the location, freely accessible LoRaWAN networks may be available via community networks such as The Things Network [21]. If there are no public LoRaWAN gateways in the deployment area, the user can set up their own gateway. While the coverage of open LoRaWAN networks is not as good as the 4G coverage, we opted against any mobile networks that require a monthly subscription fee.

The sensor PCB is designed around an ESP32-S3 microcontroller. The ESP32-S3 marks a good trade-off between efficiency, cost, and processing power. Compared to its predecessor (ESP32), the S3 microcontroller is much more suitable for machine learning and digital signal processing [22]. The S3 has an optimized instruction set for matrix multiplications and other operations important to digital signal processing and machine learning inference. Combined with kernel optimizations for TensorFlow, these optimizations lead to seven times faster inference on Espressif’s inference example [23]. These improvements benefit our sensor’s sound event classification. The microcontroller further leaves sufficient headroom for computing additional metrics and other processes, such as battery management. The RP2040 (found, for example, in the Raspberry Pi Pico) has also been considered, but has no direct support for external PSRAM. The RP2040 provides only 264 KB of on-chip RAM, which is insufficient for the sound event classification model used in this work. The newer RP2350 would be another potential alternative, but was not widely available at the time of designing this sensor.

Two reasons led to the decision to design a custom PCB, as opposed to off-the-shelf development boards. First, while off-the-shelf development boards are easy to source, they do not contain all required components. Therefore, multiple PCBs would have to be combined, resulting in unnecessarily high wiring effort and increased size. Secondly, the custom PCB is optimized in terms of energy efficiency, which is very important in this use case due to the required power independence. If one still decides to replicate this sensor using off-the-shelf development boards, the power consumption may be higher, and the firmware may need to be adjusted slightly.

Besides the microcontroller, the custom sensor PCB hosts several additional integrated circuits (ICs): It contains a dedicated Real Time Clock (RTC) for time keeping, which is independently powered by a coin cell. While the microcontroller can keep track of time as well, it is less accurate: With the RTC (specifically DS3231SN), the time drift is limited to 1 min per year (±2.5 ppm) [24]. The microcontroller itself would only provide an accuracy of ±5–11 min per year (±10–20 ppm), depending on the exact configuration and use of sleep modes [25], [26]. The PCB also contains a solar charge controller, a microSD card slot, a LoRaWAN chip (used to connect to the LoRaWAN network), and an integrated 868 MHz antenna used for LoRaWAN. Lastly, two current and power monitor ICs monitor solar charging and battery consumption. The backside of the PCB connects the battery holders.

The microphone (TDK ICS-43434) is mounted on a separate PCB, allowing it to be mounted away from the main hardware enclosure. This MEMS microphone has a sensitivity tolerance of ±1 dB, making it suitable for noise monitoring purposes. The microphone's digital I2S interface avoids potential inaccuracies from analog signal transmission.

### 2.1 Firmware components

**Fig. 2:**
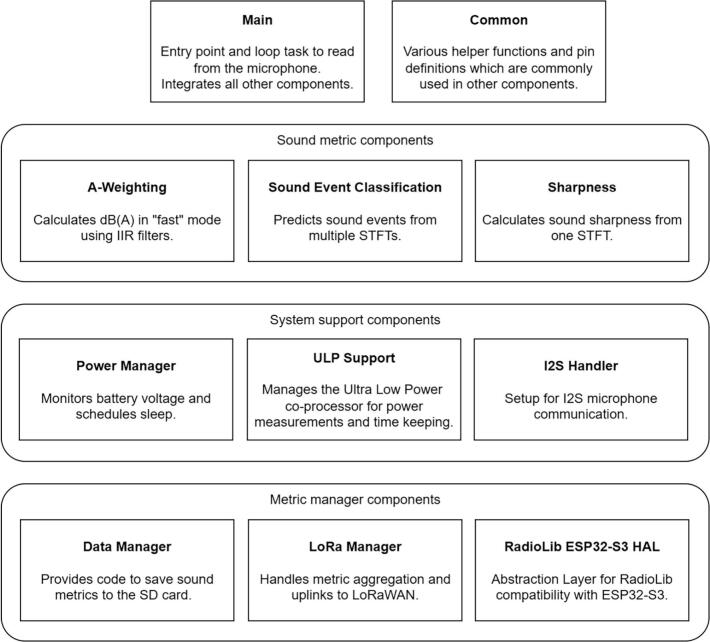
Firmware components grouped into sound metrics, system support, and metric management components.

Fig. 2 shows the firmware components of the sensor. The system has been made modular to make software extensions and changes easier. The main component serves as the application entry point and starts all other components. It runs a loop task that continuously reads from the microphone and feeds the audio stream to the relevant components. The so-called common component defines the GPIO pins specific to the custom sensor PCB and contains various helper functions to reduce redundant code.

#### 2.1.1 Sound metric components

The A-weighting, sound event classification, and sharpness component are grouped together as sound metric components.

The A-weighting component is based on an existing open-source project [10]. We adjusted the code to be compatible with the ESP32-S3. The component uses Infinite Impulse Response (IIR) filters on chunks of 6,000 amplitude samples to calculate A-weighted sound pressure levels in dB(A). This results in eight measurements per second due to the sampling frequency of 48 kHz (48,000/6,000 = 8). Eight measurements per second are common in sound level meters, which implement the IEC 61672–1 fast mode or mode F [27]. The sensor does not apply any time-weighting.

The sound event classification module can detect the presence of the following sound sources: vehicles, honking, aviation, sirens, human chatter, dog bark, bird song, church bells, music, wind, and rain. In order to do so, the module first creates Mel spectrograms from short-term Fourier transforms (STFT) over 3-second intervals. After each interval, the corresponding Mel spectrogram is forwarded to a convolutional neural network (CNN). The CNN inference is executed in a separate process on the second CPU core to ensure that all measurement processes can continue without interruption (the classification takes approximately 800 ms). In previous research [28], nine CNN models were compared regarding their accuracy and efficiency. Out of these models, the sensor uses the CNN, which achieved the highest Macro F1 score. It consists of 281,147 trainable parameters, which are distributed over five convolutional layers and a hidden dense layer. The model has been trained on 65,815 sound samples from various datasets and reached a Macro F1 score of 65.69 % and a precision of 76.27 %. For further information on the model and training data, we kindly refer to our previous work [28].

Lastly, the sharpness component calculates acoustic sharpness, a measure of the spectral distribution of sound energy that increases with higher-frequency content. [29]. For example, squeaking brakes have high sharpness, while low-bass sounds result in lower sharpness. Higher sharpness is perceived as less pleasant. The component merges sharpness calculated from three STFTs, each with a hop size of 1024 samples, which equates to roughly 16 measurements per second. To reduce computational demands and, consequently, power consumption, the component uses the same STFTs as the sound event classification. Therefore, sharpness is not equivalent to implementations that follow other standards, such as Zwicker sharpness. Zwicker sharpness is based on specific loudness measured in sones, while our STFT implementation is based on A-weighted loudness. It is not recommended to directly compare sharpness measures obtained by different standards.

If needed, further sound metric components can be implemented.

#### 2.1.2 System support and metric management components

The power manager component monitors the battery voltage, which is continuously retrieved through the Ultra Low Power (ULP) co-processor. If the battery is low, the sensor will periodically stop monitoring to balance power consumption with the power generated by the solar panel. The power manager component is responsible for this process.

The ULP Support component contains ULP co-processor code and acts as a bridge between both processors, and therefore exchanges the data required for the power manager component. The ULP also tracks current flow in and out of the battery, as well as the current generated by the solar panel. Future versions of the power manager might utilize this information for alternative power management algorithms.

The I2S handler initializes the I2S connection to the microphone, which is required to read the audio stream. As previously mentioned, the sampling frequency is set to 48 kHz.

Lastly, the data manager, LoRa manager, and RadioLib ESP32-S3 HAL save metrics to the microSD card and send aggregated metrics via LoRaWAN to the cloud. If not required, the user can disable the LoRaWAN uplinks. For the microSD card, we designed a sensor-specific binary file format to minimize the file size. The user can make use of a Python package to convert these binary files into DataFrames or CSV files. The file format and Python package will be further discussed in the operating instructions.

### 2.2 Intended purpose

The sensor system allows researchers to:

• Monitor noise pollution and soundscapes without the need for a permanent power supply.
• Increase the spatial resolution with more measurement locations due to the low cost of the devices.
• Conduct sound event classification on the sensor while ensuring privacy by design, as no audio needs to be recorded or transmitted.
• Modify the open-source firmware for other purposes, such as predicting soundscape indices or metrics for biodiversity research.

While the primary focus is on temporary deployments over a few months, the system can also be configured as a permanent sensor due to its LoRaWAN connectivity. For prolonged deployments in humid environments, it is recommended to use a different solar panel without a USB port due to possible corrosion. Instead, cables should be soldered directly to the solar panel.

## 3 Design files summary

**Table:** 

Design file name	File type	Open source license	Location of the file
Primary sensor board KiCad project and production files	Electronics design files	MIT	https://osf.io/56txz/files/
Primary sensor board schematics	PDF	MIT	https://osf.io/76cfp
Microphone PCB KiCad project and production files	Electronics design files	MIT	https://osf.io/s8dtu/files/
Microphone PCB schematics	PDF	MIT	https://osf.io/xwk5n
Solar panel holder print files	STL/OBJ file for 3D printing	MIT	https://osf.io/2bn7v/files/
Firmware source code and binary	Source code files	GNU GPL v3	https://osf.io/zmjry/files/
SSCM reader	Source code files	GNU GPL v3	https://osf.io/63ug8/
Assembly instructions video	video	MIT	https://osf.io/n3m4k

The design files can broadly be categorized into production files, KiCad project files, CAD files, and source code. Production files are needed to manufacture the primary PCB and the microphone PCB. These files can be found in the “production/” subfolders of the corresponding component. KiCad project files and schematics can be used as hardware documentation. The KiCad project files are also the starting point for hardware adjustments. The files related to the solar panel holder can be used to print one of the holder variants (wall mount or pole mount). The firmware is written in C++ and assembler. The firmware component contains the source code and compiled binary files. Lastly, a video shows the steps to assemble the sensor. These steps are also explained in this paper.

## 4 Bill of materials summary

**Table:** 

Designator	Component	Number	Cost per unit −currency	Total cost − currency	Source of materials	Material type
Primary PCB	See separate BOM in repository	1	20.00 €	20.00 €	https://osf.io/56txz/files/ghvfr	Other
Microphone PCB	See separate BOM in repository	1	20.00 €	5.00 €	https://osf.io/s8dtu/files/xgjhv	Other
Solar panel	Monocrystalline solar panel 6 W maximum power (Pmax), 6 V at Pmax (Vmp). Dimensions without connector: 275 x 170 x 2 mm	1	11.50 €	11.50 €	https://cutt.ly/prBhUxqR	Semiconductor
Enclosure	Waterproof Plastic Box Enclosure. Dimensions: 140x82x38mm	1	3.00 €	3.00 €	https://cutt.ly/DrBhAGzO	Polymer
PLA Filament	380 g PLA	1	3.80 €	3.80 €	https://cutt.ly/NrBhSUmu	Polymer
18,650 battery	Samsung INR18650-33G 3150 mAh	1–2	3.20 €	3.20–––6.40 €	https://cutt.ly/OrBhFpdB	Other
RTC 2032CR battery	CR2032 3 V Lithium Button Cells	1	0.40 €	0.40 €	https://cutt.ly/krBhHJfa	Other
Windscreen	Foam windscreen. Dimensions: 3cmx2.2 cm. Hole diameter: 0.75 cm	1	0.10 €	0.10 €	https://cutt.ly/ErBhKo4t	Polymer
DuPont wire female to female	Jumper cable DuPont female to female. Length 30 cm	5	0.13 €	0.65 €	https://cutt.ly/frBhLZfi	Other
MicroSD card	Patriot Memory LX Series Micro SD card 16 GB − PSF16GMDC105	1	3.39 €	3.39 €	https://cutt.ly/2rBhZk7d	Other
M2.5 spacers	M2.5 spacer 20 mm standoff	4	0.10 €	0.40 €	https://cutt.ly/HrBjnRqB	Other
M2.5 screws	M2.5 screw 10 mm length	4	0.05 €	0.20 €	−	Other

Detailed BOMs for the primary PCB and the microphone PCB can be found in the accompanying repository (see also design files summary).

## 5 Build instructions

The build procedure consists of the following steps:

1. Assembly of the primary PCB
2. Assembly of the microphone PCB
3. 3D printing of a solar panel holder
4. Assembly of the system
5. Flashing firmware and configuration

For visual assembly instructions, please refer to the assembly video linked in the design files summary. The following subsections outline the individual steps and explain the overall system design to facilitate future modifications.

### 5.1 Assembly of the primary PCB

**Fig. 3:**
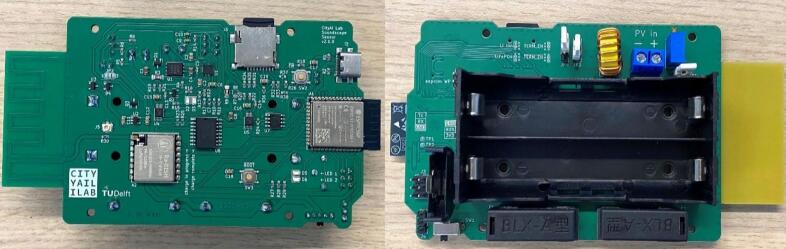
Front and back view of the primary PCB.

The front of the primary PCB contains the microcontroller, solar charger, real-time clock (RTC), LoRaWAN module, and an SD card holder. The main battery holder and RTC battery holder are both connected to the backside (see Fig. 3). The PCB also includes an integrated LoRaWAN antenna. Small SMD parts are tedious to solder by hand. All such components have been placed on the front of the PCB. Therefore, it is strongly recommended to order the PCBs with the front side pre-assembled to reduce manual work (e.g., via JLCPCB or PCBWay). The backside consists of larger though-hole parts, which are easy to solder manually using a conventional soldering iron.

Please note that the integrated LoRa antenna is inferior to external antennas in terms of signal range. For this reason, the PCB has an IPEX connector for a dedicated antenna. If the user wishes to connect one, it is recommended to remove R30, a zero-ohm resistor that connects the integrated antenna.

The primary PCB has two jumpers related to the battery configuration. One specifies if Li-Ion or LiFePo4 batteries are used, while the other one can be used to enable or disable charger termination. Charge termination should be disabled, as this would prevent the board from being powered directly by solar if the batteries are fully charged. We merely provide the option for charge termination for potential unforeseen future use cases.

#### 5.1.1 Tuning the solar charging circuit

The efficiency of solar panels varies based on the voltage at which they operate [30]. The voltage at which a solar panel generates maximum power is called maximum power voltage (V_mp_). Solar charging circuits typically ensure that the solar panel operates close to Vmp through a mechanism known as Maximum Power Point Tracking (MPPT). This sensor uses a constant voltage MPPT method with a configurable target V_mp_. Different solar panels may have different values of V_mp_. Consequently, the charging circuit must be tuned to match the V_mp_ of the connected solar panel. Otherwise, the sensor may not charge at all, or charge at lower efficiency and thus, slower.

The solar charging circuit is tuned via a potentiometer on the back of the PCB. To perform the tuning, Jumper one must be shortened, and its voltage (V_jumper_) must be measured with a multimeter. Then, the sensor needs to be supplied with the target V_mp_ on the solar input (V_in_). The solar panel recommended in our bill of materials has a V_mp_ of 6  V. We recommend using an adjustable power supply to ensure the correct voltage. Now, the potentiometer should be adjusted until the jumper voltage (V_jumper_) equals 1.2 V.

If an adjustable power supply is not available and the user has no means to provide the desired V_mp_ to V_in_, then an alternative tuning process can be used. Any known voltage can be supplied to V_in_, but the target for V_jumper_ will be different and needs to be calculated:

(1) $$\begin{array}{c} V_{\mathrm{jumper}}=\frac{V_{\mathrm{in}}}{V_{\mathrm{mp}}}\times 1.2 \end{array}$$

Please note that the sensor only charges if the power switch is turned on, because the switch completely disconnects the batteries from the rest of the board. This design decision has been made to minimize safety hazards.

#### 5.1.2 Preliminary test of PCB

Before mounting the PCB into the enclosure, it is recommended to verify its basic functionality. When connected to the computer via the USB-C port, the sensor should be detected as a serial device.

After disconnecting the USB cable, insert at least one 18,650 battery, a fuse, and a CR2032 coin cell battery. Now the user must turn on the sensor for a few minutes to observe any potential abnormal behavior. While no firmware has been installed yet, a faulty PCB could still lead to a short circuit, and therefore, excessive heat or a burned fuse. If no abnormal behavior is observed, the user can power off the device and continue with the assembly process.

### 5.2 Assembly of the microphone PCB

The sensor uses an ICS-43434 microphone on a separate, circular PCB. Separating the microphone from the main PCB allows minimizing reflections from nearby surfaces and allows the installation of a wind muff. The ICS-43434 is selected because it has a guaranteed sensitivity of ±1 dB. The microphone is connected digitally via I2S, which is natively supported by the ESP32 microprocessor family. Due to guaranteed accuracy and digital communication, it is not necessary to calibrate each sensor unit individually, which saves time and effort during the assembly process.

We strongly recommend the microphone PCB assembly at the factory to ensure correct handling. Manual assembly is challenging and may alter the microphone characteristics due to excessive force or heat. If soldered manually, a soldering oven, soldering hot plate, or heat gun is required. A soldering iron alone does not suffice.

We decided against the use of existing ICS-43434 breakout boards, as (at the time of designing the sensor), no round breakout board was available. Available breakout boards would make it difficult to attach a wind muff and may be more prone to water damage. The custom breakout board further uses SMD pin headers, making the PCB more water-resistant than through-hole pin headers. Through-hole pin headers could result in a short circuit if the outside of the microphone PCB is exposed to water. If one strictly wants to avoid ordering a custom PCB for the microphone, then a breakout board such as “Adafruit I2S MEMS Microphone Breakout − ICS-43434″ can be used with some modifications to the microphone mounting.

### 5.3 3D printing of the solar panel holder

The solar panel holder can be printed using the STL file in the repository. It is designed for the solar panel and sensor enclosure listed in the bill of materials. If a solar panel or enclosure of different dimensions is used, the solar panel holder needs to be adjusted. For this purpose, a Shapr3D design file is in the repository. For other programs, such as Blender, an FBX version is also available.

We provide two solar panel holder designs depending on the available 3D printing build volume. The full-sized solar panel holder requires a build volume of at least 18.5 cm (L) × 4.5 cm (W) × 27.5 cm (H). Note that many standard-sized 3D printers don’t fulfill the height requirement. A shortened solar panel holder (21.5 cm) is available, but it is less aesthetically pleasing, as the solar panel is sticking out at the side. We recommend PLA as a filament as it is easier to print than PETG or ABS.

Once the holder is printed, the solar panel can slide into the solar panel holder. We recommend using a small zip tie to ensure the solar panel stays in place.

### 5.4 Assembly of the system

Once the components above are acquired, they can be put together with the sensor enclosure. This step requires a screwdriver, drill, and heat gun.

**Fig. 4:**
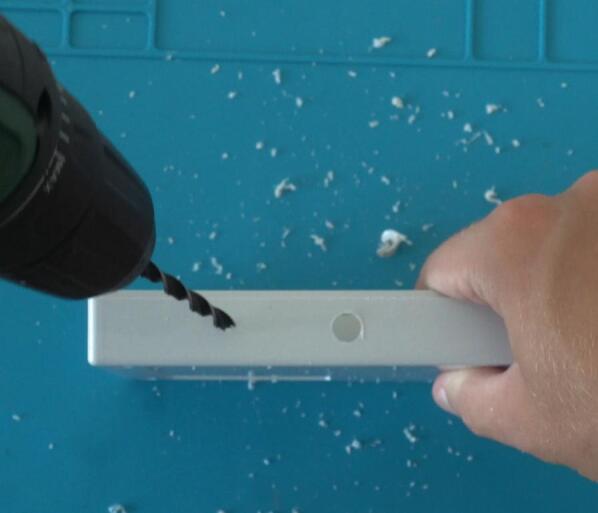
Drilling of sensor holes.

**Fig. 5:**
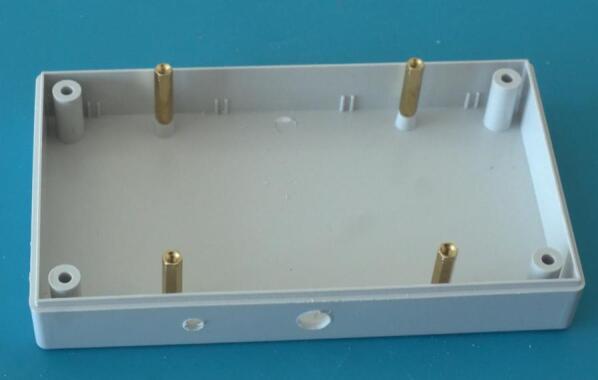
Placement of M2.5 spacers.

Begin by drilling two holes in the enclosure: One for cables connecting the microphone and the other for cables connecting the solar panel (see Fig. 4). Ensure to drill into the correct side of the enclosure, namely the side that has screw holes on the outside. On the same side, install the M2.5 spacers (Fig. 5). We will later place the primary PCB on these spacers.

Now, prepare the microphone PCB. Start by connecting the jumper wires to the microphone PCB and note down which color connects to which pin. Make sure the cables are long enough to reach the PCB via the microphone pipe. It is recommended to use hot glue to keep the jumper wires connected to the microphone PCB. Afterwards, pull the jumper wires through the pipe, which will hold the microphone. Carefully glue the PCB onto the pipe.

**Fig. 6:**
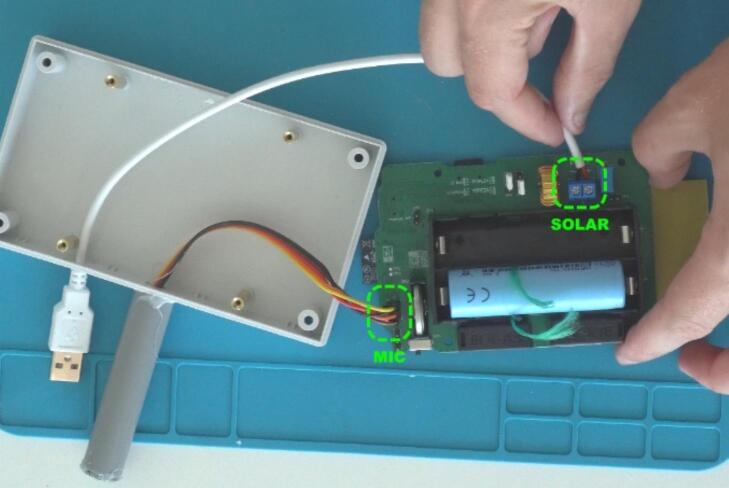
Connection of the microphone and solar power wires.

With the microphone prepared, pull its cables through the large enclosure hole in the middle of the enclosure. Then glue the microphone pipe to the enclosure. At this point, you may also push the exposed ends of the solar panel USB cable through the other enclosure hole. Connect the microphone cables and solar panel cables to the primary PCB (see Fig. 6).

Now that all cables are connected, place the primary PCB on the M2.5 spacers and gently tighten the screws. This concludes the hardware assembly. But before the enclosure can be closed and mounted, the software installation needs to be completed.

### 5.5 Firmware installation

The firmware can either be compiled from the source code and then flashed, or the compiled firmware can be downloaded directly as a binary from the repository and flashed. Compiling from source code allows modifications of the source code, while flashing the downloaded binary demands fewer software requirements.

#### 5.5.1 Compiling from source

**Fig. 7:**
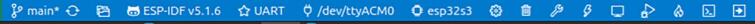
Visual Studio Code buttons from the ESP-IDF extension.

First, download the source code from the repository. We recommend cloning the GitHub repository to receive the latest changes. To compile the source code, ESP-IDF needs to be installed. A user-friendly way for this installation is to use Visual Studio Code with the ESP-IDF plugin. The plugin offers a graphical interface for the installation of all dependencies. Once everything is installed, you can compile the project by either pressing the wrench icon in Fig. 7 or searching for the command “ESP-IDF: Build your project” by pressing Ctrl+Shift+P.

Now you can proceed by flashing the firmware to the sensor.

#### 5.5.2 Flashing the compiled firmware

Before the firmware can be flashed onto the device, connect the sensor to the PC using its internal USB Type-C port. Make sure the device is turned on. If the user has compiled the firmware from source, the user can simply use ESP-IDF within Visual Studio Code. If the user downloaded the compiled firmware, the user can use esptool.py. Both approaches are explained below.

To flash the compiled firmware with Visual Studio Code, first make sure the sensor device is selected in the toolbar from Fig. 7. Select the device by pressing the socket icon or by searching for the command “ESP-IDF: Select port to use”. Depending on your operating system, the device may show up as /dev/ttyACM0, COM0, or similar. If other devices are connected, the number at the end of the device name may be higher (e.g., COM5). Once the correct device is selected, you can flash the firmware by pressing the flash button in the toolbar (lightning icon in Fig. 7). Alternatively, search for the command “ESP-IDF: Flash (UART) your project”. Now, verify the device log by pressing the screen icon (Fig. 7) or by executing the command “ESP-IDF: Monitor Device”. One should see log messages requesting the device to be configured.

To flash the compiled firmware without Visual Studio Code, first install the required Python package: “pip install esptool”. For further installation details, consult: https://docs.espressif.com/projects/esptool/en/latest/esp32s3/index.html. Before you can flash the firmware, you need to find out the serial device name. If you use Windows, you can use Device Manager to find the device name. Under “Ports” you should find an Espressif device (this is the sensor) with a name such as COM0 or similar. On Linux, you can find relevant devices with the following command: “ls /dev/ttyACM*”. If more than one device is connected, you can use ”dmesg | grep tty“ to find the Espressif device (this is the sensor). Once you have identified the device name, run the following command within the firmware folder (you need to replace the port parameter with your device name):

*esptool.py* *−-chip esp32s3 −-port /dev/ttyACM0 −-baud 460,800 write_flash 0x0 bootloader.bin 0x8000 partition-table.bin 0x10000 idf_sensor.bin*

After flashing the firmware, check that the firmware is running correctly by checking the sensor's serial output. Depending on your operating system, you can use tools like Putty (Windows) or a command line tool such as screen (Linux): “*screen /dev/ttyACM0 115200*″. You should periodically see a message requesting that the device needs to be configured. The device configuration is part of the operation instructions.

## 6 Operation instructions

This section explains how to configure the sensor, how to mount the sensor, and how to access the sensor data.

### 6.1 Configuring the sensor firmware

After the firmware is installed, the sensor needs to be configured before it starts to monitor the sound environment. During this configuration, the time will be synced with the internet. While the sensor uses a dedicated real-time clock, time will still slowly drift over time. It is therefore recommended to repeat the configuration if the sensor has not been used for a while.

The configuration further sets a device ID, which will be written to the SD card. It can therefore be used to identify which data is coming from which sensor. Optionally, the sensor can also be configured to transmit aggregated metrics via LoRaWAN.

After flashing the firmware, the device automatically enters configuration mode. The configuration mode can also be entered by holding the BOOT button for 2 s until the status LED begins to flash quickly.

The repository contains a tool for configuring the sensor, called configurator.py. Before you can use the tool, make sure the sensor is in configuration mode, connected to the computer, and that no other application is connected to the device. Thus, you may need to close the Visual Studio Code monitor, Putty, or screen command. Initiate the configuration with the following command (executed in the folder where configurator.py is located): “python configurator.py configure”. Follow the instructions given by the terminal. For a list of all commands, run “python configurator.py help”.

#### 6.1.1 Connection to the cloud via LoRaWAN

If one plans on using LoRaWAN, then the device needs to be registered in The Things Network (TTN). The Things Network is a community-driven IoT network using LoRaWAN. One can either rely on Gateways from other users in the network or install their own gateway. The latter is necessary if the sensor location does not have sufficient coverage. These instructions only concern how to set up a sensor, not a gateway.

First, an account for TTN needs to be registered. In the TTN console, a new application must be created (e.g., sound-sensors). Within the new application, the sensor can be registered as an end device. Select “Enter end device specifics manually” as the input method. Select the frequency plan based on your location: E.g., “Europe 863–870 MHz (SF9 for RX2 – recommended)” for Europe. Select specification 1.1.0 as the LoRaWAN version. Select “RP001 Regional Parameters 1.1 revision A” for the regional parameters. Note that the standard may change over time, and a different version may be required at some point in the future. Enter 16 zeros for the JoinEUI and confirm. Generate a DevEUI, DevKey, and NwkKey. Set a unique End device ID. We recommend using the same ID here as the device ID in the sensor configuration (e.g., sound-sensor-01). Finally, confirm by pressing “Register end device”. Now, the DevEUI, AppKey, and NwkKey can be copied from the TTN console when requested by the configurator.py.

### 6.2 Mounting the sensor

Before mounting the sensor, ensure the device is turned on.

**Fig. 8:**
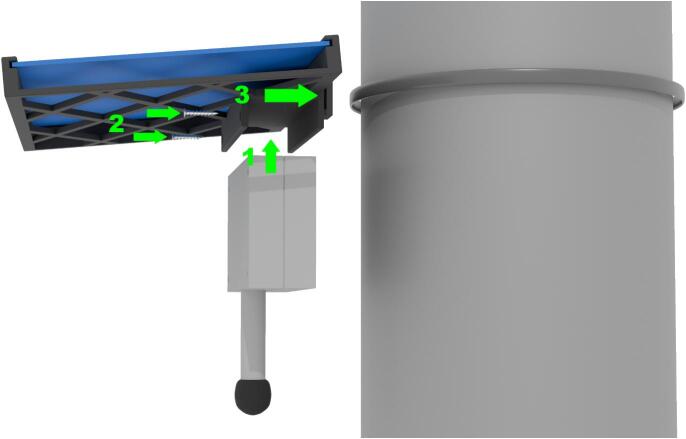
Sensor mounting on a light pole.

To mount the sensor, close the enclosure and secure the two screws on the lower side of the enclosure (the microphone side). Connect the USB power cable to the solar panel. Slide the enclosure into the solar panel holder and tighten two long screws to the two top holes of the sensor enclosure. This step is very important as the enclosure may fall out of the holder if it is not held in place with two screws. Now you can use zip ties or an adjustable mounting bracket to mount the sensor to a street light pole, sign pole, or another object (see Fig. 8).

### 6.3 Reading sensor metrics from the SD card

The sensor saves all metrics in a custom binary file format to reduce the size requirements for the SD card. In comparison, a CSV file would be approximately 10x larger to store the same information. To read the custom file format, the repository includes a Python library called sscm reader (short for soundscape metric reader). Alternatively, the most recent version of the tool will also be available via *pip install sscm_reader*. The reader can be used in a Python script and provides a function to read SSCM files. The function read_sscm returns pandas DataFrames for loudness, detected sources, sharpness, and battery voltage over time. You can find a visualization of the metrics in the validation.

### 6.4 SSCM file format specification

For normal use, the user does not need to be aware of the specifics of the file format. For completeness and potential adjustments, we still describe the format. The file header contains so-called magic bytes to make the format computer-readable. Therefore, each SSCM file starts with the following bytes: 0x00, 0x00, 0x63, 0x69, 0x74, 0x79, 0x61, 0x69, 0x5F, 0x73, 0x63, 0x5F, 0x73, 0x65, 0x6E, 0x73, 0x6F, 0x72, 0x5F, 0x76, 0x30, 0x31. In ASCII, these bytes represent the text “cityai_sc_sensor_v01”. The magic bytes are followed by 4 bytes with the file creation time as a Unix timestamp with ms resolution. This is then followed by the sensor ID and the number of labels for the sound event detection, which concludes the file header. After the header, the file contains an unspecified number of blocks, with each block representing one measured sound metric. The first byte in each block specified the metric type (e.g., dB(A) level or sound event). The metric type then prescribes the length and content of the block. Table 1 contains the specifications of all metric blocks.

**Table 1:** Metric blocks and size in SSCM file.

Metric	Total size	Content
dB(A)	13 bytes	Metric type (1 byte), timestamp (8 bytes), dB(A) value as float (4 bytes).
Sound event	53 bytes	Metric type (1 byte), timestamp (8 bytes), 11 floats for metric probabilities (4 bytes each).
Sharpness	13 bytes	Metric type (1 byte), timestamp (8 bytes), sharpness value as float (4 bytes)
Voltage	11 bytes	Metric type (1 byte), timestamp (8 bytes), mV value as int (2 bytes)
Enter sleep mode	11 bytes	Metric type (1 byte), timestamp (8 bytes), seconds to sleep as int (2 bytes)
Nightly power management	13 bytes	Metric type (1 byte), timestamp (8 bytes), monitoring ratio as float (4 bytes)

## 7 Validation and characterization

The ICS-43434 microphone has a sensitivity tolerance of ±1 dB as stated in its datasheet (condition: 1 kHz tone at 94 dB SPL). We confirmed this in tests with 10 microphones. The Acoustic Overload Point (AOP) is 120  dB SPL. Meaning that sounds up to 120 dB can be recorded without distortion. The microphone's frequency response ranges from 60 Hz to 20 kHz. Further specifications of the microphone can be found in the datasheet. The sensor drives the microphone at a sample rate of 48 kHz.

### 7.1 Lab validation

**Fig. 9:**
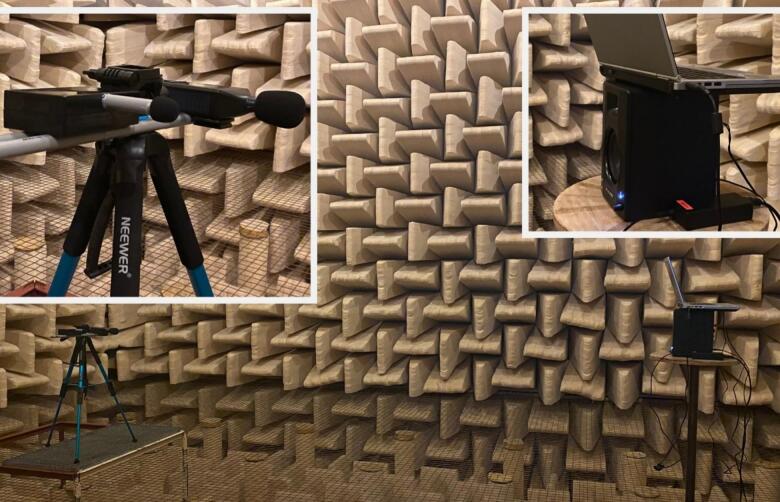
Test setup for the sensor validation in an anechoic chamber with a speaker on the right and the sensor and a reference sound level meter on the left.

We validated the dBA measurements of the sensor system in an anechoic chamber. For this, various test sounds were played using one M−Audio BX4 speaker. The sensor and a Class 2 SLM were 3.6 m apart from the speaker. The SLM measurements were used as a reference for the sensor measurements. Fig. 9 shows the test setup. The experiment has been conducted twice, once with the sensor facing downward and once with the sensor facing forward. The downward-facing configuration better reflects real-world usage, as the microphone will be pointing downward, while the forward-facing configuration matches the SLM orientation. For both configurations, multiple test sounds have been played at two different loudness levels. The first loudness level is around 82 dB at 1 kHz. The second loudness level is around 63 dB at 1 kHz. Loudness for the other test sounds varies slightly from this due to the difference in energy distribution.

**Fig. 10:**
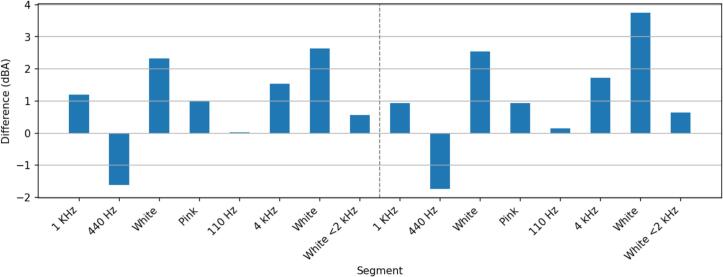
Loudness difference between reference SLM and sensor in downward-facing orientation (intended orientation).

Fig. 10 shows the deviations between the sensor measurements and the reference measurement for the downward-facing configuration. The pure tone accuracy is high with a maximum deviation of less than 2 dB for the 4 kHz tone. The deviations are similar for both the loud and medium scenarios.

**Fig. 11:**
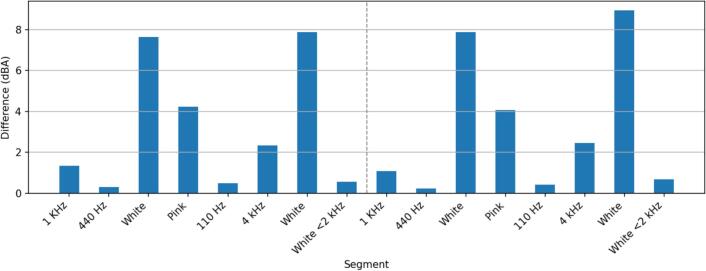
Loudness difference between the reference SLM and sensor in forward-facing orientation.

Fig. 11 shows the deviations for the forward-facing configuration. In this setup, the difference between the sensor and the SLM is larger compared to the downward-facing configuration, in particular for white noise. This may be caused by a boost of high frequencies when the sound source is parallel to the microphone, as is common with MEMS microphones. This scenario is rare in real-world deployment with diffuse sound fields and various sound sources at different relative angles to the microphone. Thus, we deem the deviations from the downward-facing test to be closer to real-world performance.

### 7.2 Power consumption

The average power consumption of the system is 60 mA during normal operation. When the sound event detection is disabled, the average current drops to 49 mA. This difference comes from the utilization of the second core and a higher clock rate during sound event detection.

The current draw is rather stable across the battery voltage range. Theoretically, the recommended 3,150 mAh battery would therefore result in 52.5 h of continued operation without any solar charging. With two batteries, the theoretical timespan is 105 h. In practice, the timespan is slightly lower due to factors such as voltage regulator inefficiency.

The solar panel has a maximum current of 1 A at the maximum power point of 6 V. Since the charge controller is programmed to charge at up to 1 A, one battery can be charged in a bit more than 3 h. In real-world conditions, the solar panel output is likely lower.

Finally, the power management component will adjust the observation-to-sleep time ratio to reach an equilibrium between ingoing and outgoing power. This adjustment occurs once per night. The observation time ratio is therefore constant for 24 h. With sufficient sunlight, the sensor monitors the sound metrics uninterrupted.

### 7.3 Conclusion

This paper describes the design and operation of a low-cost, grid-independent soundscape sensor. The system integrates a custom sensor PCB with an ESP32-S3 at its core. The PCB integrates components specific to sound sensing applications, such as LoRaWAN functionality, a microSD card slot, a solar charging circuit, and a real-time clock for accurate timekeeping. The firmware has been written in C++ without unnecessary abstraction layers to improve efficiency. The sensor measures loudness in dB(A), psychoacoustic sharpness, and detects the presence of sound events such as traffic noise, sirens, or bird song.

Currently, 39 sensors are deployed in and around Delft to analyze temporal and spatial patterns in urban noise pollution. Another ongoing deployment in a cooperation between the City of Amsterdam and the Responsible Sensing Lab aims to monitor construction site noise [31]. The Responsible Sensing Lab trained a separate ML model to detect different construction-related sound events.

## Ethics statements

Our work did not involve human subjects or animal experiments.

**Declaration of generative AI and AI-assisted technologies in the manuscript preparation process**

During the preparation of this work, the authors used ChatGPT in order to refine the manuscript. After using this tool/service, the authors reviewed and edited the content as needed and take full responsibility for the content of the published article.

## CRediT authorship contribution statement

**Lion Cassens:** Writing – review & editing, Writing – original draft, Validation, Software, Methodology, Conceptualization. **Maarten Kroesen:** Writing – review & editing, Supervision. **Simeon Calvert:** Writing – review & editing, Supervision, Conceptualization. **Sander van Cranenburgh:** Writing – review & editing, Supervision, Conceptualization.

## Declaration of competing interest

The authors declare that they have no known competing financial interests or personal relationships that could have appeared to influence the work reported in this paper.
